# Dr. Dill on Cutaneous Absorption

**Published:** 1826-07

**Authors:** 


### DISSERTATIO PHYSIOLOGICA INAUGURALIS DE INHALATIONE PER CUTEM. AUCTOR JOANNES DILL, A.M. C.M. COLL. GLASG. &c. &c. 8vo. EDINBURGI, 1825.

Dr. Dill, it appears, received his education, both philosophical and medical, in the University of Glasgow; and, during his residence at that ancient seat of learning, his diligence was unremitting, and his acquirements extraordinary. By particular reasons, however, he was induced to take his degree of M.D. at Edinburgh; and, on that occasion, he produced the Inaugural Dissertation which we shall now briefly notice. In this, he begins with stating that, "quamvis nuperiùs de absorptione per cutem a multis dubitatum est, eandemque decidere quidam etiam apertè negarunt, nihilominus hæc doctrina inter viros antiquiores multum in honore habebatur." He then proceeds, with much ingenuity and in a very philosophical manner, to bring successively under consideration, the principal hypotheses, experiments, facts, and inductions advanced, in all ages, by the asserters, as well as those by the opponents, of the doctrine of cutaneous absorption; and is led, in the course of this discussion, to observe, p. 22, "quoniam, vero, qui disciplinæ de inhalatione cuticulari adversantur, nullum argumentum, sæpiùs vel fiduciâ majori, adducunt, quam quid pondus eorum qui in balneum descendunt, immersione nihil afficiatur; experimenta aliquot, quæ ipsi ad hanc litem dirimendam instituimus, proferemus." These experiments are six in number, and go far certainly to establish the original proposition: they appear to have been contrived with much judgement and executed with great exactness; and the detail of them is remarkable for its conciseness, precision and perspicuity. From two of them which, as well as all the rest, were often repeated, with little dissimilarity of result, Dr. Dill draws authority to say, "absorptionem cutaneam haud parùm a conditionibus corporis universi diversis affici, et cum id jejunum et fatigatum quam cum plenum sit magis pollere, deducere par est." After cursorily remarking that the same observation had been made by Keil, De Garter, Rye, Hales, and Home, he asserts, p. 28, on the evidence of his own experiments and of facts adduced by Martineau, Good, and Rush, that the power of the cuticular absorbents varies at different periods in the same person. "His igitur," he says, "et multis similibus bene perspectis absorbentia, pariter ac aliud genus quodvis vasorum, vel paralysi solvi vel torpore sopire posse arbitror, ex quo fiat, ut eventus experimentorum de effectibus immersione in corporis pondus editis, factorum, quam maximé variare possint. Annotatu nihilominus dignum èst, quod in omnibus experimentis supra memoratis, exitus esset unus, quâdam exparte saltem generalis. In iis, ubi nihil ponderis accessit, constitutionis jacturam inter immersionem quam antea minorem esse semper compertum est; unde, ergo, hæc differentia ortum ducit? Anne ab inhalatione per pulmones, inter periculum factum, adaucta; an ab exhalatione per cutem imminuta? Hanc," he replies, "jacturæ mensuram diversam non ab inhalatione per pulmones adauctâ ortam, ut mihi videtur, constat." This is followed by an experiment on his own person, and a review of those published by Young, Currie, Abernethy, Seguin and Lavoisier on the question of pulmonary absorption. On the inquiry, whether cutaneous exhalation be diminished by the warm bath, he states that, according to his experience, the body acquires less weight in proportion to the degree of heat in the bath; and, after enumerating the statements of Santorius, Rye, Robinson, De Gorter, Keil, Cruikshanks, Linnings, Abernethy, Seguin, Lavoisier and Vielly, on the daily amount of perspiration, he subjoins, "ab iis documentis quæ in arenam deducta sunt, me judice, non omnino dubitari queat, quin multæ res humores circumfluentes, per cutem sub forma pariter solida ac fluida, ingredi possint." Finally, he observes that authors are not wanting, who believe in the capability of aëriform fluids, contagious particles and morbid poisons of various kinds, being communicable to the system through the instrumentality of cutaneous absorption, and modestly sums up the discussion in these words: "Hæc omnia ad hanc functionem neglectam stabiliendam et illustrandam conferunt, eamque statum pariter sanum et morbosum, plusquam multi concedere velint, afficere pro certo statuunt."

From the interesting nature of this inquiry, and the very able manner in which Dr. Dill has conducted it, in an Essay necessarily so limited, we should have felt exceedingly disposed to undertake a comprehensive exhibition of his principles and his philosophy: but, without affecting any thing like prophetic discernment, we think we can perceive internal evidence in his summary view of the question, of its being his intention not to leave it in its present almost inaccessible form. Under such impressions, therefore, we conclude this rapid sketch with assuring the zealous author of our respect for his talents and learning, and of the satisfaction we shall derive from soon meeting him in the same path of investigation, as a public competitor for the meed of literary and professional excellence.

